# Clinical outcome measures and scoring systems used in prospective studies of port wine stains: A systematic review

**DOI:** 10.1371/journal.pone.0235657

**Published:** 2020-07-02

**Authors:** M. Ingmar van Raath, Sandeep Chohan, Albert Wolkerstorfer, Chantal M. A. M. van der Horst, Jacqueline Limpens, Xuan Huang, Baoyue Ding, Gert Storm, René R. W. J. van der Hulst, Michal Heger

**Affiliations:** 1 Department of Pharmaceutics, Jiaxing Key Laboratory for Photonanomedicine and Experimental Therapeutics, College of Medicine, Jiaxing University, Jiaxing, Zhejiang, PR China; 2 Department of Plastic, Reconstructive, and Hand Surgery, Maastricht University Medical Center, Maastricht University, Maastricht, The Netherlands; 3 Department of Pharmaceutics, Utrecht Institute for Pharmaceutical Sciences, Utrecht University, Utrecht, The Netherlands; 4 Department of Dermatology, Amsterdam University Medical Centers, University of Amsterdam, Amsterdam, The Netherlands; 5 Department of Plastic, Reconstructive and Hand Surgery, Amsterdam University Medical Centers, University of Amsterdam, Amsterdam, The Netherlands; 6 Medical Library, Amsterdam University Medical Centers, University of Amsterdam, Amsterdam, The Netherlands; Zagazig University, EGYPT

## Abstract

**Background:**

Valid and reliable outcome measures are needed to determine and compare treatment results of port wine stain (PWS) studies. Besides, uniformity in outcome measures is crucial to enable inter-study comparisons and meta-analyses. This study aimed to assess the heterogeneity in reported PWS outcome measures by mapping the (clinical) outcome measures currently used in prospective PWS studies.

**Methods:**

OVID MEDLINE, OVID Embase, and CENTRAL were searched for prospective PWS studies published from 2005 to May 2020. Interventional studies with a clinical efficacy assessment were included. Two reviewers independently evaluated methodological quality using a modified Downs and Black checklist.

**Results:**

In total, 85 studies comprising 3,310 patients were included in which 94 clinician/observer-reported clinical efficacy assessments had been performed using 46 different scoring systems. Eighty-one- studies employed a global assessment of PWS appearance/improvement, of which -82% was expressed as percentage improvement and categorized in 26 different scoring systems. A wide variety of other global and multi-item scoring systems was identified. As a result of outcome heterogeneity and insufficient data reporting, only 44% of studies could be directly compared. A minority of studies included patient-reported or objective outcomes. Thirteen studies of good quality were found.

**Conclusion:**

Clinical PWS outcomes are highly heterogeneous, which hampers study comparisons and meta-analyses. Consensus-based development of a core outcome-set would benefit future research and clinical practice, especially considering the lack of high-quality trials.

## Introduction

Port wine stains (PWS) are congenital vascular malformations that occur in approximately 0.3–0.9% of infants [1–3]. Lesions initially present as flat, red-to-pink patches and darken and thicken with age [4]. PWS are most frequently located in the face and neck and can cause functional impairment [5], skin and soft tissue hypertrophy [5], and glaucoma [6], as well as substantial psychosocial morbidity [7–9]. Despite therapeutic developments, complete PWS resolution remains rare [10–12].

Valid, relevant, and reliable outcome measures are required to accurately gauge treatment effects and compare treatment protocols and therapeutic interventions. Moreover, standardization of outcome measures is imperative for enabling comparisons between studies. Increased awareness of the importance of (high-quality) outcome measures has led to a rise in outcome measure research, especially in dermatology [13] (in particular for psoriasis [14], atopic dermatitis [15], and more recently vitiligo [16]).

Recently we have performed an analysis of clinical outcomes in PWS trials published since 1986 [10]. This review was limited in its scope because of the large variety of clinical scoring systems. The outcome measure heterogeneity has also precluded data syntheses in previous PWS meta-analyses [17,18]. Nevertheless, few studies have devoted attention to PWS outcome measures and no study has systematically analyzed the use of PWS outcome measures. Therefore, the primary goal of this study was to systematically map and analyze the use of clinical PWS outcome measures (i.e., an observer-, clinician-, or patient-reported visual assessment of treatment efficacy) in prospective PWS studies since 2005. Additionally, the use of other outcome measures in these studies was assessed. Finally, the methodological quality of included studies was investigated using the Downs & Black risk of bias checklist. The results of this study could help to inform the future development of a standardized clinical scoring system for PWS and to improve the quality of PWS research.

## Methods

This systematic review was reported in accordance with the Preferred Reporting Items for Systematic Reviews and Meta-Analyses (PRISMA) statement [19]. The review protocol was registered in the international register of systematic reviews (PROSPERO; reg. no. CRD42018115343) [20].

### Search strategy

A medical librarian (JL) performed a comprehensive, systematic search in OVID MEDLINE, OVID Embase, and the Cochrane Central Register of Controlled Trials (CENTRAL) to identify prospective PWS studies from 2005 to May 4^th^ 2020. This starting year was used to gather a sufficiently large and representative sample of current practice in clinical PWS research. The search consisted of controlled terms (i.e., MeSH terms in MEDLINE) and free-text words for PWS combined with a methodological search filter for prospective studies, including randomized controlled trials (S1 Table). The retrieved records were imported and de-duplicated in ENDNOTE X7 (Thomson Reuters, New York, NY, USA). The included studies were screened for additional relevant cited or citing references.

### Study selection and data extraction

Full-text, original studies with a therapeutic intervention performed in PWS patients and with a form of clinician-, observer-, or patient-reported clinical efficacy assessment as study outcome were included. Clinical efficacy assessment was defined as a visual evaluation of appearance or improvement without the aid of an (objective) instrument. No language restrictions were applied. Studies that exclusively included patients with syndromic forms of PWS (e.g., Sturge-Weber syndrome, Klippel-Trénaunay syndrome, etc.) were excluded. We found that the outcomes in small case series (less than five PWS patients), letters, conference abstracts, short reports, and study protocols in international trial registries were, in general, described with insufficient detail. These hits were therefore excluded. Titles, abstracts, full text versions of selected studies, and reference lists of included studies were screened independently by two investigators (IvR, SC) using Rayyan [21]. In case of disagreement between the two reviewers, a third reviewer (CvdH) was consulted until consensus was reached.

Characteristics of the outcome measure, measurement instrument, scoring system, interventions, study design and population, assessors, blinding of outcome assessment, statistical analysis, follow-up duration, the reporting of results, country of the first author, and the registration of adverse events were extracted independently by two investigators (IvR, SC). Uncontrolled or non-comparative studies (essentially case series) encompassed one and the same treatment in all patients. Controlled studies employed one or more control-groups, i.e., these compared one treatment with another form of treatment and/or placebo. Different treatments could be assigned to different patients (between-patient controlled study design) or to different sites within the same patient and/or PWS (within-patient controlled design). Discrepancies between data extractors were discussed until consensus was reached.

### Appraisal of study quality

To assess the methodological quality at the study level, a critical appraisal was performed independently by two authors (IvR, SC) using a modified version of the Downs and Black checklist [22–24]. This validated checklist consists of 27 questions regarding reporting, internal validity, external validity, and power and has been used for both randomized and non-randomized controlled studies. Additional details of this analysis are described in the supplement (S3 Appendix). In addition to the factors assessed in the Downs and Black checklist, we assessed a few additional aspects related to outcome assessment: 1) the number of outcome assessors; 2) their professional background; 3) whether outcomes were assessed based on photographs, and if so; 4) whether an attempt was made to standardize these photographs.

### Data analysis

Outcome measures were classified according to: domain [25], assessor (clinician-, observer-, parent-, or patient-reported), qualitative vs. quantitative, relative (i.e., scoring systems with a single measurement that constitutes the difference in pre-treatment and post-treatment appearance) vs. static (i.e., scoring systems that require repeated pre- and post-treatment measurements with the same scoring tool to calculate a (change) score), and global (i.e., a single-item assessment) vs. multi-item (i.e., separate assessment of two or more PWS characteristics, such as PWS border, texture, and color) measures. The data were presented using descriptive statistics (frequencies and proportions).

## Results

### Study characteristics

**Fig 1:**
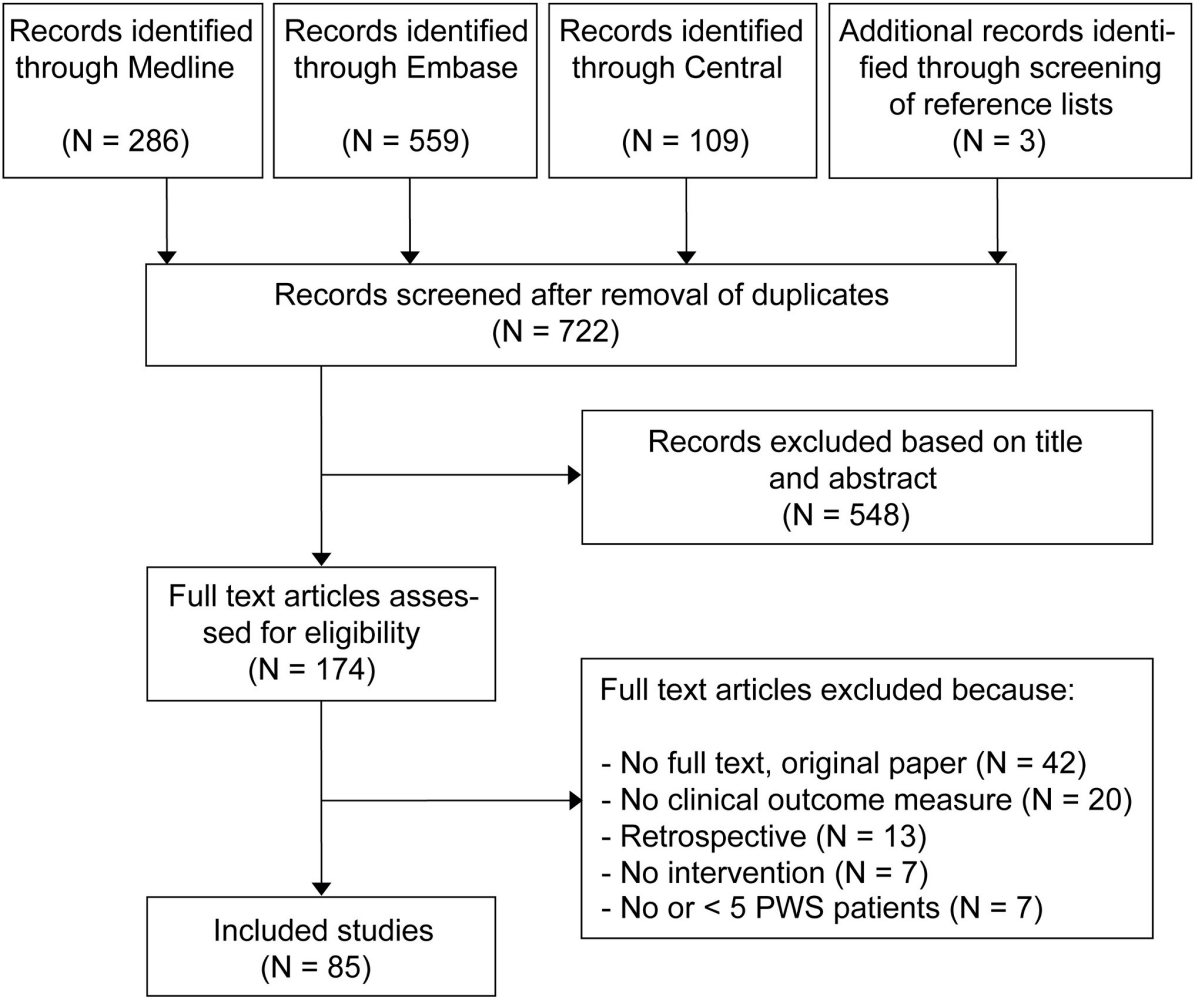
PRISMA flow diagram showing the study selection and exclusion process. In total, 85 prospective studies with a clinical outcome measure published since 2005 were included. Abbreviations: *PWS*, port wine stain.

**Fig 2:**
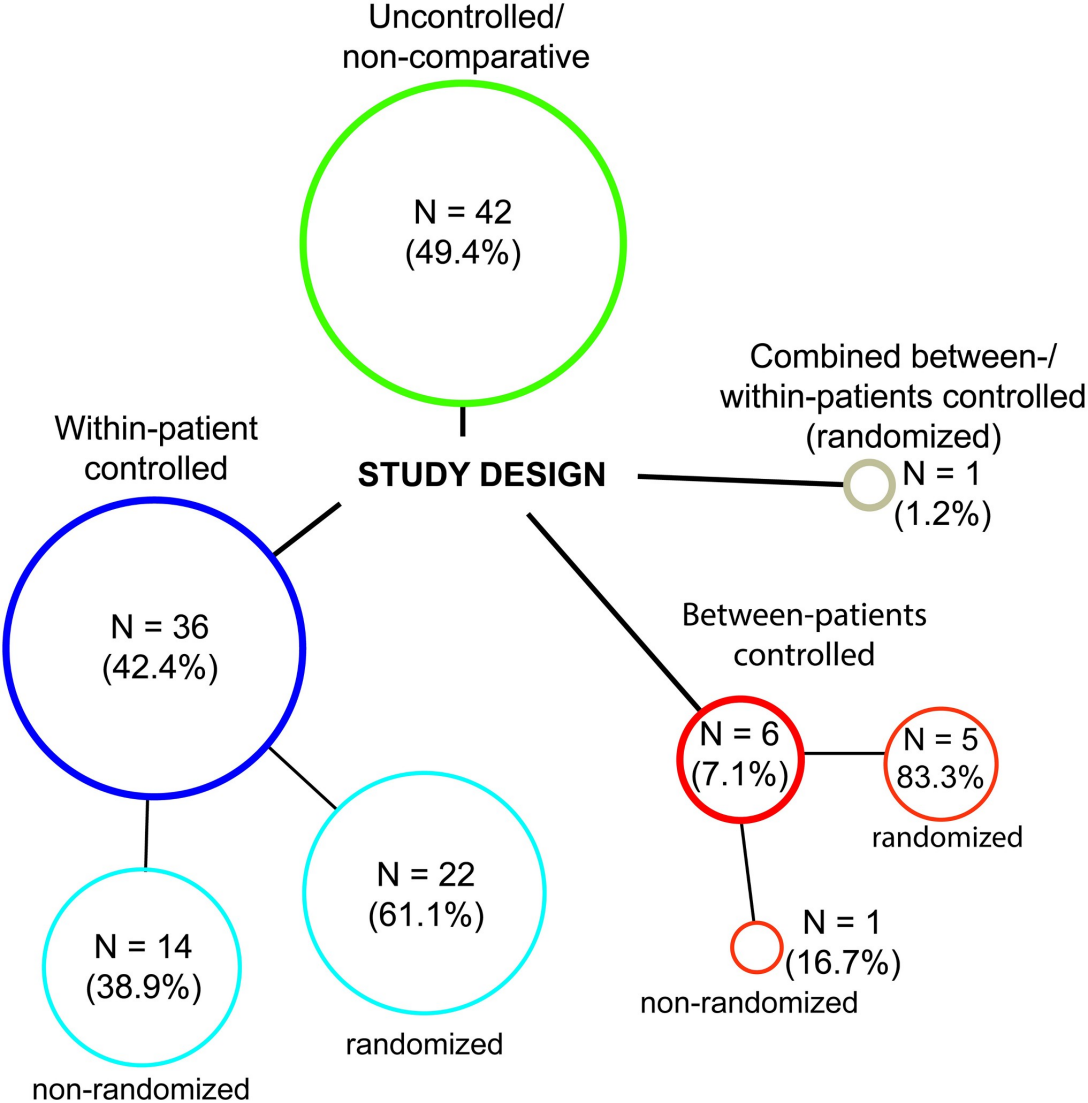
Stratification of the study designs used in the included studies. Studies with a control group (controlled studies) allocated different treatments to different treatment sites within individual patients (within-patient controlled) or to different patients (between-patients controlled).

An overview of the study selection and exclusion process is shown in the PRISMA flowchart in Fig 1. Twenty prospective studies were excluded because they did not feature a clinical outcome measure. In total, 85 studies comprising 3,310 PWS patients were included [11,26–109]. The study characteristics are presented in S2 Table and the study designs are depicted in Fig 2. The (weighted) mean age was 23.0 years and 59.7% of patients was female (S2 Table). Most studies were performed in China (N = 32) and Europe (N = 25) (S2 Table). Of the controlled studies [11,26–62,70,81,92,103,109], 30 compared two or more therapeutic interventions (e.g., PDL versus Alexandrite laser or skin cooling versus no cooling), whereas 13 studies compared treatment settings or protocols (e.g., different pulse durations, spot sizes, end points, or treatment intervals). Five studies (5.9%) were placebo-controlled. Thirty-two percent (N = 28/85) of studies was randomized (i.e., randomization of treatments to different patients or to different sites within one patient and/or PWS) [11,31,36,39,41–59,61,62,81,92,103]. Of the uncontrolled (noncomparative) studies (N = 42/75 [63–69,71–80,82–91,93–102,104–108]), 11 were performed to correlate, develop, or validate an instrument or analysis technique (such as laser speckle imaging; intended for measuring lesion characteristics, treatment effects, or efficacy), 1 study investigated different clinical assessment methods, and 1 study correlated PWS characteristics and demographic parameters with treatment results. A majority of studies (60.5%) had a minimum follow-up duration of less than 2 months (S2 Table).

### Study outcomes

**Fig 3:**
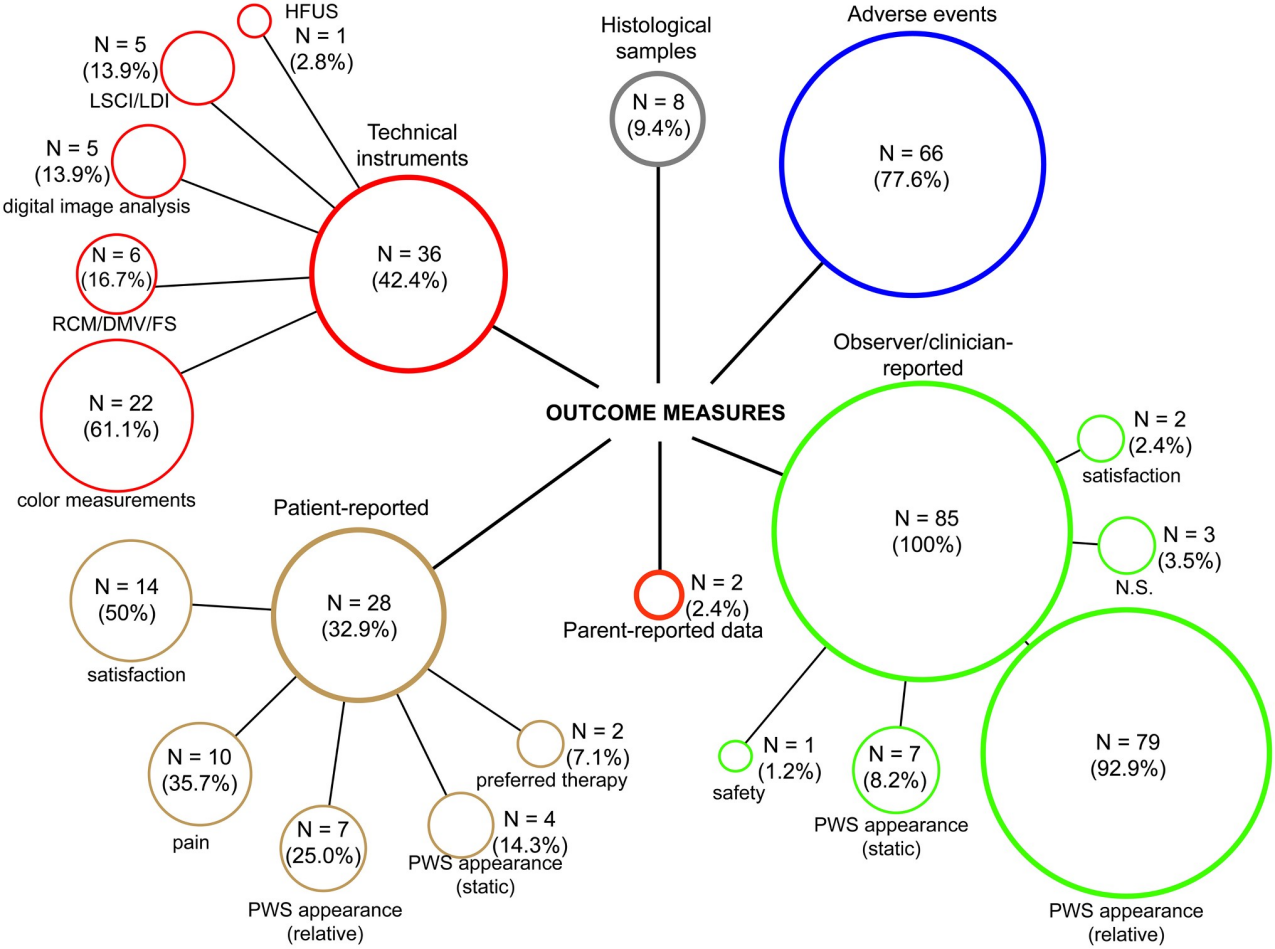
Stratification of all outcome measures. Note that the secondary percentages are relative to the primary variable and that their sum can exceed 100% as single studies assessed multiple outcomes (e.g., some studies used both a relative and static measure of treatment efficacy). Abbreviations: *DMV*, depth measuring videomicroscopy; *FS*, fluorescence spectrometry; *HFUS*, high-frequency ultrasound; *LDI*, laser Doppler imaging; *LSCI*, laser speckle contrast imaging, *NS*, not specified; *PWS*, port wine stain; *RCM*, reflectance confocal microscopy.

An overview of all study outcomes is presented in Fig 3. Only studies with a clinical efficacy assessment were included in our analysis. PWS were assessed predominantly with relative (rather than static) measures for both patient- and clinician/observer-reported data (Fig 3). Clinician/observer-reported satisfaction was included in 2 studies (2.4%). Patient-reported outcomes were measured in 32.9% (N = 28/85) of studies, which included satisfaction, PWS improvement, and treatment preference (Section Patient-reported and parent-reported outcome measures).

In addition to clinical assessment, 36 studies (42.4%) used objective instruments to objectively measure treatment efficacy or quantify other factors, such as dermal blood flow reduction. Histological samples to assess photothermally-induced changes and epidermal damage were collected in 8 studies (9.4%).

Of all studies, 77.6% (N = 66/85) systematically collected data on the presence of adverse or side effects. In one study a 4-point scoring system was used to score “safety”, i.e., the occurrence and degree of hypopigmentation or hyperpigmentation and hypertrophic or atrophic scarring [75]. Another study classified the degree of crusting based on a 3-point scoring system (‘thick’, ‘thin’, or ‘none’) [32].

### Observer/clinician-reported outcome measures and scoring systems

Inasmuch as several studies employed 2 forms of clinical efficacy assessment, a total of 94 observer/clinician-reported clinical efficacy assessments were performed. The scoring systems were not specified in 3 studies. In the remaining studies, 46 different scoring systems were employed (Table 1). Most studies (N = 79/85) used a relative measure as the primary outcome. For relative measures, a global assessment of PWS improvement (also referred to in studies as ‘blanching’, ‘lightening’, and ‘clearance’) was the most prevalent. In a majority of studies (N = 66/81) the global assessment was reported quantitatively as a percentage improvement and was categorized into subgroups (usually quartiles, which were supplemented by additional strata of 0% (N = 23/63) and/or 100% clearance (N = 5/63) in some studies). Alternatively, qualitative scoring systems were used that varied from 2 to 5 grades (Table 1). A multi-item assessment using relative scoring systems was used in 2 studies.

**Table 1:** Measures and scoring systems used for observer/clinician-reported efficacy assessments in prospective PWS trials from 2005 to May 2020.

	No. of studies (%)
**Comprehensive (global) assessments:**	
***Relative measures***	
*Quantitative scoring systems*	
0–100% (continuous) clearance/blanching	2 (2.4)
0–100% (in 5% increments) lightening	2 (2.4)
0–100% (in 10% increments) clearance/improvement	3 (3.5)
0–25%, 26–50%, 51–75% or 75–100%	6 (7.1)
0%, 1–25%, 26–50%, 51–75%, or 76–100%	4 (4.7)
‘Poor’ (0–25%), ‘fair’ (26–50%), ‘good’ (51–75%) or ‘excellent’ (76–100%)	8 (9.4)
‘Unsatisfactory’ (0–25%), ‘medium/average’ (25–49%), ‘good’ (50–74%), or ‘perfect’ (75–100%)	1 (1.2)
‘Minimal’ (0–25%), ‘fair’ (26–50%), ‘good’ (51–75%) or ‘excellent’ (76–100%)	2 (2.4)
‘Failure’ (0–24%), ‘mild’ (25–49%), ‘moderate’ (50–74%), ‘good’ (75–94%), or ‘excellent’ (>95%)	1 (1.2)
‘Failure’ (0%), ‘mild’ (1–25%), ‘moderate’ (26–50%), ‘good’ (51–75%) or ‘excellent’ (76–100%)	2 (2.4)
‘No improvement’ (0–25%), ‘mild improvement’ (26–50%), ‘moderate improvement’ (51–75%) or ‘significant improvement’ (76–100%)	1 (1.2)
‘No improvement’ (0%), ‘poor/bad’ (1–25%), ‘fair’ (25–50%), ‘good’ (50–75%) or ‘excellent’ (75–100%)	10 (11.8)
‘No improvement’ (0%), ‘poor’ (1–25%), ‘moderate’ (25–50%), ‘good’ (50–75%) or ‘excellent’ (75–100%)	1 (1.2)
‘No clearance’ (0%), ‘slight clearance’ (1–25%), ‘moderate clearance’ (25–50%), ‘good clearance’ (51–75%) or ‘excellent/very good clearance’ (>75%)	3 (3.5)
‘Grade 0’ (scarring), ‘grade 1’ (no improvement), ‘grade 2’ (0–25%), ‘grade 3’ (25–50%), ‘grade 4’ (50–75%), ‘grade 5’ (75–100%) or ‘grade 6’ (100%)	1 (1.2)
‘Worsening’ (-1), ‘no change’ (0), ‘0–25% lightening’ (1), ‘26–50% lightening’ (2), ‘51–75% lightening’ (3), ‘76–99% lightening’ (4) or ‘complete clearance’ (5)	1 (1.2)
‘No change after treatment’ (0%), ‘mild improvement’ (1–24%), ‘some improvement’ (25–49%), ‘moderate improvement’ (50–74%), ‘significant improvement’ (75–99%), or ‘complete clearance’ (100%)	1 (1.2)
‘No improvement’ (0%), ‘mild/slight improvement’ (1–25%), ‘moderate improvement’ (26–50%), ‘marked/much improvement’ (51–75%), or ‘near complete or complete clearance’ (>75%)	4 (4.7)
‘No improvement’ (0–20%), ‘some improvement’ (20–60%), ‘great improvement’ (60–90%) or ‘almost cured’ (≥90%)	1 (1.2)
‘No improvement’ (0–20%), ‘some improvement’ (20–59%), ‘great improvement’ (60–89%) or ‘nearly completely resolved’ (≥90%)	2 (2.4)
‘Ineffective’ (0–20% clearance), ‘improvement’ (20–59%), ‘response’ (60–89%) or ‘complete response’ (> 90%)	1 (1.2)
‘No efficacy’ (the color was mostly unchanged in the treated area; 0–20%), ‘alleviation’ (the color partially faded in the treated area; 20–60%), ‘good efficacy’ (the color significantly faded in the treated area; 60–90%), or ‘cured’ (the color mostly faded in the treated area; ≥ 90%)	3;1^a^ (4.7)
‘No improvement’ (0–30%), ‘mild improvement’ (31–60%), ‘moderate improvement’ (61–90%) or ‘significant improvement, nearly cured’ (91–100%)	1 (1.2)
‘Minimal lightening’ (~25%), ‘obvious lightening’ (25–50%), ‘slight residual color’ (50–75%) or ‘became normal skin’ (75–100%)	1 (1.2)
‘Effective’ (partial depigmentation in the treatment area; ≥ 20% improvement) or ‘ineffective’ (color unchanged or mostly unchanged in the treatment area (< 20% improvement)	1 (1.2)
‘No significant change’ (0%), ‘minimal lightening/result not remarkable’ (25%), ‘obvious lightening/ somewhat remarkable result’ (50%), ‘slight residual color’ (75%) or ‘appears as normal skin’ (100%)	2 (2.4)
0–10 VAS (0 = worsening or no improvement at all, 10 = complete clearance)	1 (1.2)
0–10 VAS (0 = normal skin, 10 = dark-red color)	1^a^ (1.2)
-1 (worsening), 0 (no change), 1 (slight improvement), 2 (moderate improvement), 3 (marked improvement) or 4 (complete clearance)	1 (1.2)
*Qualitative scoring systems*	
Cosmetic appearance is ‘superior’ or ‘comparable’ to other test sites	1^a^ (1.2)
‘Darker’, ‘no change’, ‘lighter’	1 (1.2)
‘Poor’, ‘moderate’, ‘good’ or ‘excellent’	1 (1.2)
‘Ineffective, ‘moderate’, ‘good’ or ‘excellent’	2^a^ (2.4)
‘Poor/unchanged’, ‘moderate’, ‘good’ or ‘excellent’	1 (1.2)
‘No difference’, ‘mildly improved’ or ‘greatly improved’	1 (1.2)
‘Moderate’ (disappearance of about 30% of the lesion), ‘good’ (disappearance of almost 70% of treated vessels) and ‘excellent’ (disappearance of PWS)	1 (1.2)
‘Excellent’ (color is close to normal skin color and no scar formation), ‘good’ (marked blanching, thicker lesion become flat, no scar formation), ‘fair’ (partial blanching, thicker lesion becomes moderately flat), ‘poor’ (slight blanching, thicker lesion becomes slightly flat) or ‘no change’	3 (4.7)
‘No or minimal improvement’, ‘fair improvement’, ‘good improvement’ or ‘excellent improvement’ (total clearance or almost total clearance)	1 (1.2)
‘No change’, ‘minimal lightening’, ‘obvious lightening’, ‘slight residual color’ or ‘became normal skin’	1 (1.2)
***Static measures***	
Redness 0–10 VAS (0 = redness of normal skin, 10 = maximum redness)	1 (1.2)
Cosmetic appearance 0–10 (0 = very bad cosmetic appearance; 10 = very good cosmetic appearance)	2^a^ (2.4)
Munsell color chart score	2 (2.4)
**Multi-item assessments:**	
***Relative measures***	
Skin color, skin texture, and overall clinical outcome were assessed separately on a 1–4 scoring system (1 = no signs of skin change associated with PWS, 4 = significant change in skin associated with PWS). Change in overall outcome was converted to a percentage improvement.	1 (1.2)
Efficacy, purpura and homogeneity were each assessed and classified into ‘better with’ or ‘better without’ the study intervention	1 (1.2)
***Static measures***	
Skin color (1 = light pink, 2 = pink, 3 = dark pink, 4 = red, 5 = light purple, 6 = purple, 7 = dark purple) and texture level grading (‘flat’, ‘hypertrophic’, ‘cobbled’ or ‘other’)	1^a^ (1.2)
A modified Patient and Observer Scar Assessment Scale (POSAS) score (items: ‘vascularity’, ‘pigmentation’, ‘thickness’, ‘relief’, ‘pliability’, and ‘surface area’), extended with ‘overall opinion of the skin’ and ‘habitual bleeding’	1^a^ (1.2)
**Scoring system not specified**	3 (3.5)

Both global (single-item) and multi-itemed (several, individually scored characteristics) PWS scoring systems are shown. These were divided into qualitative vs. quantitative, and relative (a single measurement score that compares pre- and post-treatment) vs. static (the difference between repeated pre- and post-treatment scores) measures.

^a^These outcomes were used as secondary outcome. Abbreviations: *PDT*, photodynamic therapy; *PWS*, port wine stain; *VAS*, visual analogue scale.

In a few studies with relative measures as the primary outcome, a secondary efficacy outcome was included in the form of another relative (and global) measure (N = 5/85) or a static measure (N = 4/85). Static measures included the Patient and Observer Scar Assessment Scale (POSAS), a 10-point scoring systems for ‘redness’ or ‘cosmetic appearance, the (decrease in) scores on a Munsell color chart, and multi-itemed assessment of skin color and texture. Two studies also utilized dermoscopy-derived outcomes (using an unspecified scale or the intraoperative observation of vascular rupture; not included in the analyses).

**Fig 4:**
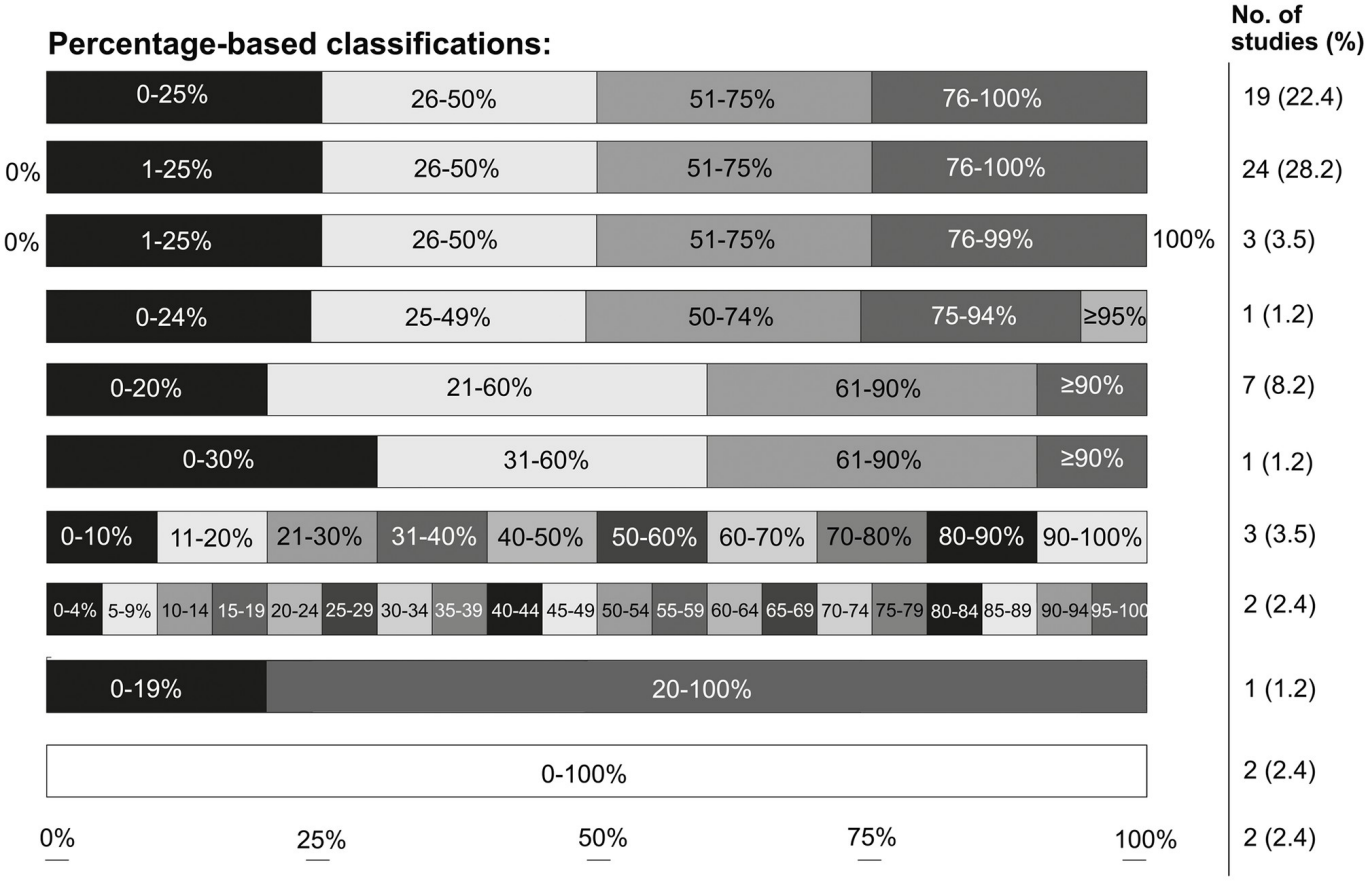
Scoring systems used to classify observer/clinician-reported percentage ‘improvement’, ‘lightening’, ‘clearance’ or ‘blanching’ for global assessment of port wine stain improvement. The percentage-based scoring systems in Table 1 were stratified according to their categories.

Although most studies used a classification based on percentage improvement, the differences in the number of subgroups and subgroup ranges (shown in Fig 4) complicated study comparison. Numerous studies also used inconsistent and contradictory (mathematical) statements to describe subgroups. In total, 26 different scoring systems based on percentage blanching (or percentage ‘improvement’, ‘lightening’, or ‘clearance’) were identified in 65 studies. The data of a maximum of 57.4% of efficacy assessments (N = 54/94) could theoretically be converted into one common, simplified classification of quartile percentages (i.e., 0–25%, 26–50%, 51–75%, and 76–100%). However, many studies failed to report the scores of each category or only reported mean scores for the entire cohort, which precluded actual pooling of the data. Consequently, the data of maximal 43.5% of studies (N = 37/85) could be pooled into one uniform scoring system.

Observer/clinician-reported satisfaction was included as a secondary outcome in two studies (using a 0–4 scoring system or an ‘ineffective, ‘moderate’, ‘good’, or ‘excellent’ score).

### Patient-reported and parent-reported outcome measures

Satisfaction with treatment (N = 14/85) was the most commonly included patient-reported outcome and was measured using 1 of 9 different scoring systems (Fig 3 and Table 2). Patient or parent-reported PWS improvement was included in 13 studies (15.3%) using 1 of 11 different scoring systems (similar to scoring systems used for clinician/observer-reported assessment). Ten studies assessed patient-reported pain. Patient-preferred treatment (for patients that underwent 2 or more forms of treatment) was included in 6% of within-patients controlled studies (N = 2/36). No studies measured quality of life or measures of functioning.

**Table 2:** Patient- and parent-reported outcome measures and scoring systems in prospective trials from 2005 to May 2020.

	No. of studies (%)
**Patient satisfaction:**	
0–10	4 (4.7)
0–10 VAS (0 = poor, 10 = excellent)	1^a^ (1.2)
0–100%	1 (1.2)
0–100	1 (1.2)
4-point scoring system (0 = not satisfied, 3 = extremely satisfied)	2 (2.4)
‘Not satisfied’, ‘slightly satisfied’, ‘moderately satisfied’, ‘satisfied’ or ‘very satisfied’	1 (1.2)
‘Not satisfied’, ‘little satisfied’, ‘somewhat satisfied’, ‘satisfied’ or ‘very satisfied’	1 (1.2)
‘Poor’ (not satisfied at all), ‘fair’ (slightly satisfied), ‘good’ (moderately satisfied), or ‘excellent’ (very satisfied)	1 (1.2)
‘Ineffective’, ‘moderate’, ‘good’ or ‘excellent’	1 (1.2)
NL	1 (1.2)
**Patient-reported PWS improvement:**	
‘No response’ (0%), ‘slight response’ (<25%), ‘moderate response’ (25–49%), ‘good response’ (50–74%) or ‘very good response’ (75–100%)	1 (1.2)
‘No clearance’ (0%), ‘slight clearance’ (< 25%), ‘moderate clearance’ (25–50%), ‘good clearance’ (51–75%), or ‘excellent clearance’ (> 75%)	2 (2.4)
‘No improvement’ (0%), ‘slight improvement’ (> 0%–25%), ‘moderate improvement’ (> 25%–50%), ‘much improvement’ (> 50%– 75%) or ‘near complete or complete remission’ (> 75%–100%)	1 (1.2)
1–4 (1 = poor, 2 = moderate, 3 = good, 4 = very good)	1 (1.2)
‘Ineffective’, ‘moderate’, ‘good’ or ‘excellent’	2 (2.4)
‘No change’, ‘mild’, ‘moderate’ or ‘significant’ improvement	1 (1.2)
Cosmetic appearance 0–10 (0 = very poor/bad cosmetic appearance, 10 = very good cosmetic appearance)	2 (2.4)
Redness score 0–10 VAS (0 = redness of normal skin, 10 = maximum redness)	1 (1.2)
A modified Patient and Observer Scar Assessment Scale (POSAS) score (items: ‘pain’, ‘itching’, ‘color’, ‘thickness’, ‘stiffness’, and ‘irregularity’), extended with ‘overall opinion of the skin’ and ‘habitual bleeding’	1 (1.2)
**Parent-reported PWS improvement:**	
Change in size, overall satisfaction with the results, change in color, wish to continue therapy (score 1–5)	1 (1.2)
−1 to 5 (−1, worsening; 0, no change; 1, less than 25% lightning; 2, 26% to 50% lightening; 3, 51% to 75% lightening; 4, 76% up to 99% lightening; 5, complete clearance	1 (1.2)
**Treatment preference**	1; 1^a^ (2.4)
**Pain:**	
Wong-Baker Faces scale (0–10 VAS)	1 (1.2)
0–10 VAS (0 = no pain, 10 = extremely severe pain)	1 (1.2)
0–10 VAS	2 (2.4)
VAS	1 (1.2)
1–10 (1 = slight pain, 10 = strong pain)	1 (1.2)
0–100	1 (1.2)
1–10 (1 = mild pain, 10 = severe bee sting-like pain)	1 (1.2)
0–10 VAS (0 = no pain, 10 = unbearable)	1 (1.2)
0–10, in comparison to other site	1 (1.2)

^a^Assessment for pediatric patients was performed by parents. Abbreviations: *NL*, not listed; *PWS*, port wine stain; *VAS*, visual analogue scale.

### Objective measures using optical instruments and digital image analysis

Non-invasive, objective assessment using optical instruments or digital image analysis techniques was used in 42.4% of studies (N = 36/85). The techniques employed were colorimetry (N = 12/85), reflectance spectrophotometry (N = 10/85), digital image analysis (N = 5/85), laser speckle contrast imaging (N = 4/85), fluorescence spectrometry (N = 2/85), depth measuring videomicroscopy (N = 3/85), laser Doppler imaging (N = 1/85), high-frequency ultrasound (N = 1/85), and reflectance confocal microscopy (N = 2/85). The outcomes obtained with these instruments are listed in Table 3.

**Table 3:** Outcomes of objective instruments.

Optical instrument	Outcome	No. of studies (%)
Depth measuring videomicroscopy	Vessel number, diameter, and depth	3 (3.5)
Laser speckle contrast imaging	Dermal blood flow/tissue perfusion change	4 (4.7)
Laser Doppler imaging	Skin perfusion	1 (1.2)
Spectrophotometers	Δa* and ΔE	1 (1.2)
	Erythema and vascularity indices	1 (1.2)
	Erythema index	4 (4.7)
	Change in skin reflectance	1 (1.2)
	Difference in reflected light at 585 nm between affected and unaffected skin	1 (1.2)
Fluorescence spectrometer	Therapeutic effect correlation index (TECI)	1 (1.2)
Digital photography	ΔE	1 (1.2)
	Peak signal-to-noise ratio analysis	1 (1.2)
Cross-polarized photography with a calibrated diffuse R standard to quantify RGB channels	Relative changes in G:B image channel pixel values ratio	1 (1.2)
	Erythema index	1 (1.2)
VISIA-CR system (polarized photography)	Erythema index	1 (1.2)
Colorimeter	Δa*, ΔE and blanching rate	4 (4.7)
	ΔE and blanching rate	5 (5.9)
	ΔE and ΔE in comparison to pre-treatment lesion	1 (1.2)
	Δa*	1 (1.2)
	Erythema index	2 (2.4)
Reflectance confocal microscopy	Vessel diameter and density	2 (2.4)
	Mean vessel depth	1 (1.2)
	Relative blood flow	1 (1.2)
High-frequency ultrasound	Presence of linear hypoechoic signal, dermal density and thickness	1 (1.2)

Δa* is the change in redness (a*) using the L*a*b* color system as determined by the Commission Internationale de l’Eclairage (CIE). ΔE refers to the color difference according to the CIE76 formula [110], usually in comparison to normal (contralateral) skin.

### Methodological quality of prospective PWS studies

**Fig 5:**
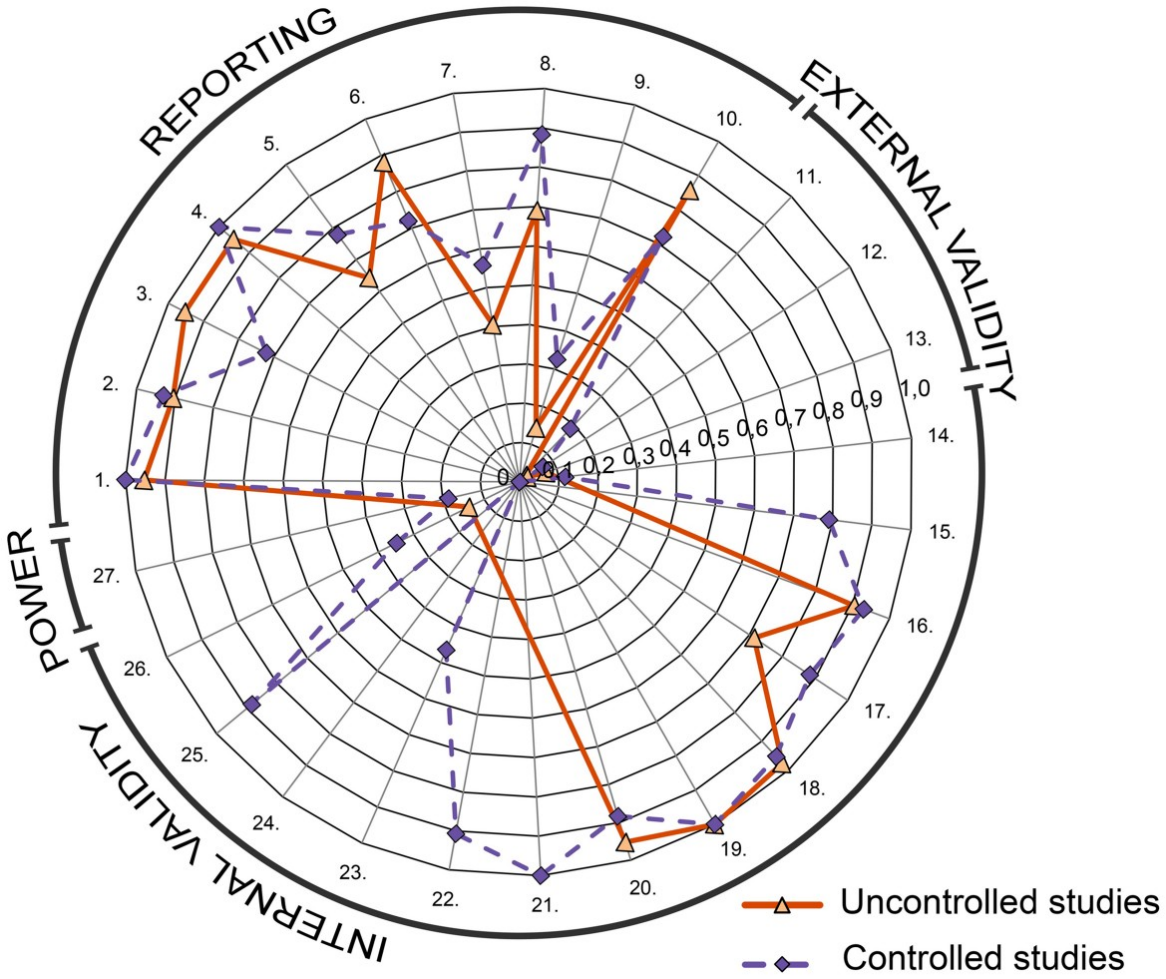
Mean Downs and Black checklist scores per item for controlled and uncontrolled studies. Item numbers are indicated within the outer ring. For uncontrolled studies, items 14, 15, 21–25, and 27 were considered irrelevant and were therefore omitted. All scores are normalized to 1 inasmuch as one item (#5) has a maximum score of 2 instead of 1.

**Fig 6:**
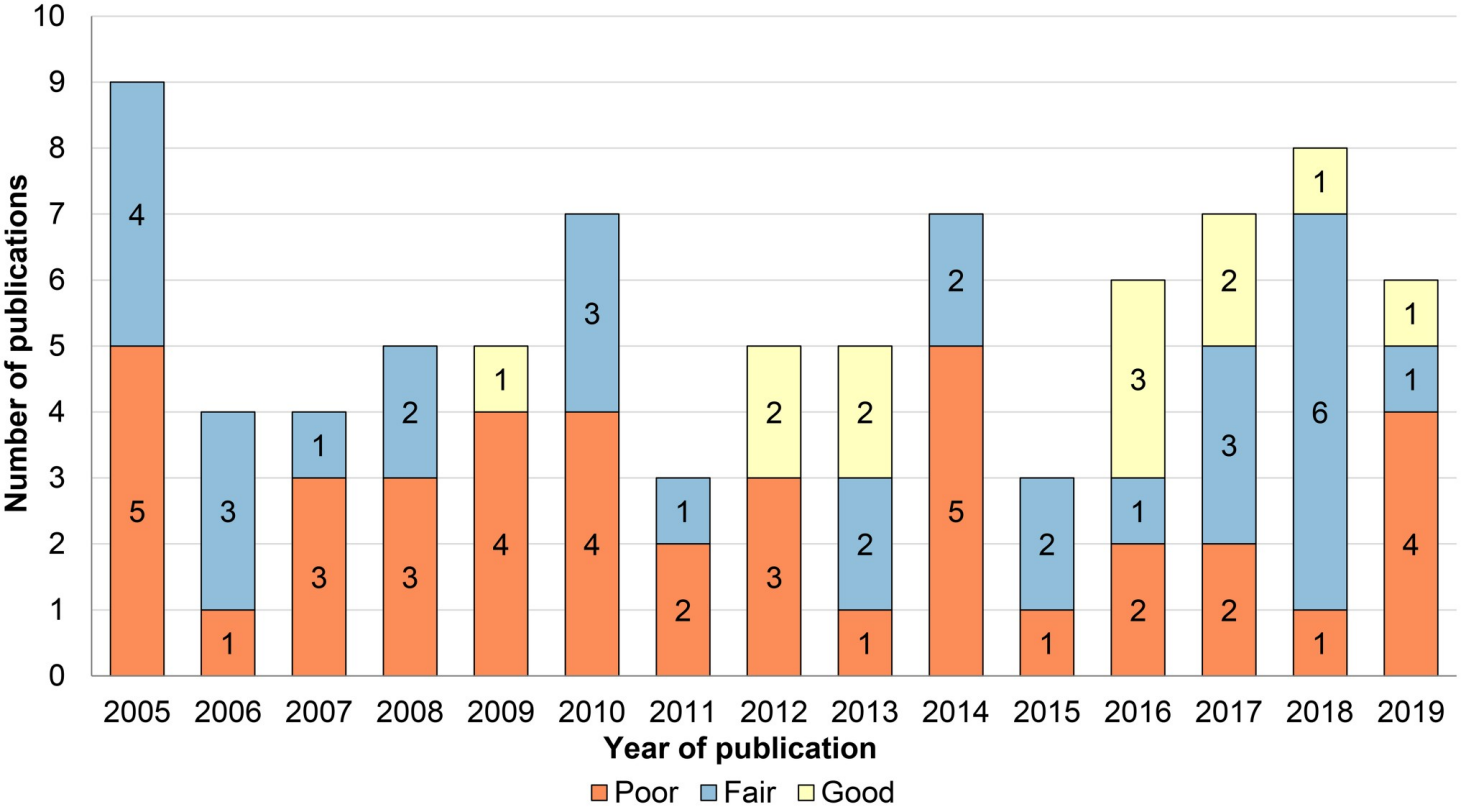
Trends in quality of published studies. The stacked columns show the absolute annual number of published studies stratified by Downs and Black checklist scores. The year 2020 (N = 1 ‘good’ quality study) was omitted because it is incomplete. No studies of ‘excellent’ quality were found.

The mean (± SD) Downs and Black risk of bias checklist score was 15.3 ± 4.0 (18.0 ± 3.3 for controlled and 12.4 ± 2.4 for uncontrolled studies; S1 Checklist). Studies were of good (N = 13), fair (N = 31), and poor (N = 41) quality. No excellent studies were found. The mean score per item for controlled and uncontrolled studies is presented in Fig 5. The items related to ‘external validity’ were scored particularly poor. In controlled studies, the items 9, 11–14, 24, 26, 27 were not satisfied most frequently. In uncontrolled studies most points were lost in items 7, 9, 11–13, and 26. In the studies of good quality a ‘percentage improvement’ scale was most frequently used as the primary outcome (92.3%, N = 12/13). Fig 6 shows the annual number and quality of published PWS trials, which suggests a small positive trend in good quality studies.

**Fig 7:**
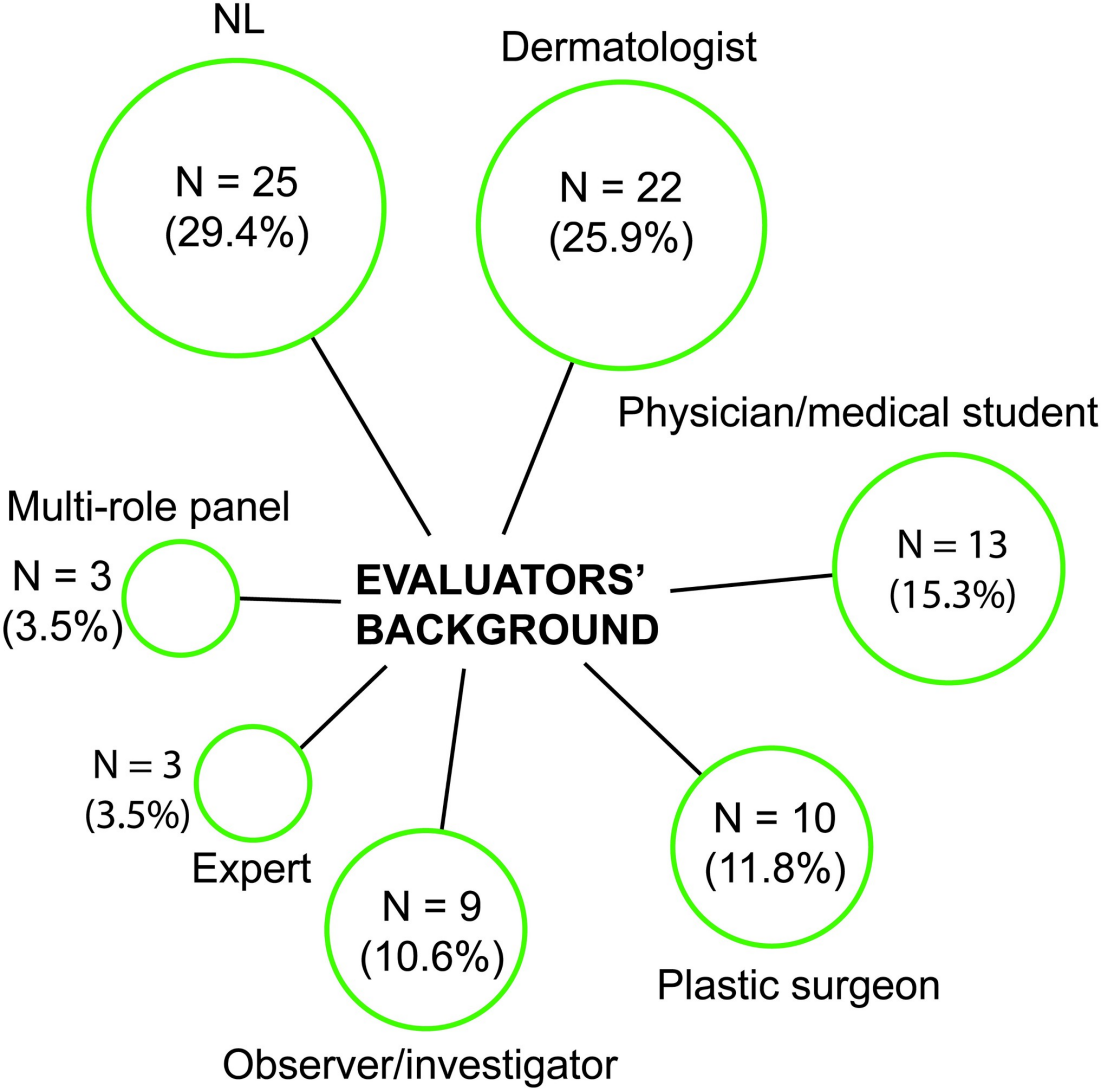
The professional background of the evaluator who performed the primary efficacy assessment. Three studies used a panel of evaluators with different professional backgrounds (compositions were: a physician and a dermatologist; a plastic surgeon and a dermatologist, and a plastic surgeon, medical student, and investigator). Studies with ‘experts’ did not specify the experts’ background. Abbreviations: *NL*, not listed.

In most studies primary clinical efficacy assessment was performed by multiple evaluators (N = 52/85; 61.2%). Evaluation was reportedly performed in a blinded manner in 54.1% (N = 46/85) of all studies and 69.4% (N = 34/49) of controlled studies. The professional background and distribution of the evaluator(s) that performed clinical efficacy assessment is shown in Fig 7. In 41.2% of studies (N = 35/85) a dermatologist or plastic surgeon was involved. In 29.4% (N = 25/85) of studies the professional background was not reported. Of all studies, 87.1% (N = 74/85) reported the use of photographs to assess treatment efficacy. These were reported to be taken under standardized conditions in 54.1% (N = 40/74).

## Discussion

Our systematic analysis revealed considerable heterogeneity in clinical outcome measures and scoring systems in prospective PWS studies. Most studies used a global (observer/clinician-reported) efficacy assessment with percentage improvement as primary outcome measure. Due to the variations in scoring systems and score conversions (e.g., reporting only the mean improvement for the entire patient cohort), only 44% of studies (N = 37) had a clinical outcome that could be included in one common, simplified scoring system to enable inter-study comparative analysis. Other scoring systems included multi-item assessment of PWS and the difference in repeated pre- and post-treatment appearance scores. Almost half of all studies based treatment efficacy outcomes, additionally, on an objective measurement, such as colorimetry. Nevertheless, even in these studies there is a gamut of differential outcome measures. Patient-reported outcomes were included in a minority of studies and included pain, PWS improvement, and satisfaction.

In the past two decades, good outcome measures for clinical studies, i.e., those that are valid, consistent, accurate, reproducible, and error-free, have increasingly been recognized as essential elements for evidence-based clinical decision making. As a result, study end-points have come under increasing scrutiny [111]. Concurrently, efforts have been made to standardize trial outcomes and thereby enable meta-analysis and other forms of data pooling, e.g., by developing an agreed minimum set of outcomes known as a ‘core outcome set’ (facilitated by the ‘Core Outcome Measures in Effectiveness Trials (COMET) Initiative [112]) and, in dermatology, the establishment of the International Dermatology Outcome Measures (IDEOM) initiative [113,114] and the Cochrane Skin Group—Core Outcome Set Initiative (CSG-COUSIN) [115]. By mapping the outcome measures currently in use, this review could aid in the development of a core outcome set for PWS.

In 1992, Pickering et al. reviewed the assessment methods used to assess the response of PWS to laser treatment and found substantial variability [116]. The authors pointed to the subjective nature of visual assessment and advocated the use of noninvasive optical instruments, such as colorimetry, reflectance spectrophotometry, and Doppler flowmetry to objectively quantify PWS improvement. This has since been reiterated several times by others [117–120]. Meanwhile, the objective scoring methods have been expanded and now include digital image analysis [90,118,121], reflectance confocal microscopy [122], optical coherence tomography [123,124], depth measurement videomicroscopy [125], laser speckle contrast imaging [63,126], and spatial frequency domain imaging [127]. Interestingly, these tools do not always correlate with visual assessment [81,128], underscoring potential flaws in subjective assessment tools. On the other hand, the final goal of treating PWS is to improve visibility and noticeability rather than change objective measures, such as dermal blood volume or blood flow, so this should be reflected in study outcomes. Moreover, most of these (optical) instruments are costly and not clinically available. In an altogether different approach, Lanigan proposed to use differences in pre- and post-treatment patient morbidity or satisfaction as study outcomes [129], which closely aligns with the increasing recognition of the importance of patient-reported outcomes [130]. In our sample, patient-reported outcomes were included in only 32.9% of studies, none of which involved measures of quality of life or functioning. The objective assessment versus patient-based subjective approach raises an important question as to what is most important clinically: the actual degree of PWS blanching or the patients’ perception of therapeutic efficacy (or perhaps even patients-perceived PWS-related life-impact). Regardless of the abovementioned objective assessment methods, (subjective) clinical efficacy assessment remains the most popular approach in prospective PWS studies as evidenced by the results of our systematic analysis (i.e., our study was limited to studies with a clinical assessment but only 20 studies were excluded for not complying with this criterion). Despite past efforts to standardize PWS study outcome measures, clinical scoring systems have remained highly variable.

Our review also showed that the quality of prospective PWS studies is generally poor inasmuch as no excellent and only thirteen good quality studies were found. Low Downs and Black scores was mostly attributable to incomplete disclosure of the patient recruitment process and patients lost to follow up, the randomization process, and the lack of patient blinding. It is likely that scores have been influenced to some extent by poor reporting (rather than poor study practice and design), which could be aggravated by the word limits imposed by dermatological journals. Also, the scores for uncontrolled studies are curbed to some extent due to the inclusion of studies without the primary goal to evaluate intervention effect (N = 12/42, section Study Characteristics).

Our systematic analysis was limited to prospective studies. However, there are no indications that retrospective studies perform any better in terms of outcome homogeneity. Another limitation is the fact that only studies with a clinical assessment were included. This means that some outcomes, particularly technical outcomes, may have been missed. The authors considered the assessment period of over 15 years sufficiently representative of current practice.

Consensus on the best outcome measures for PWS studies is lacking, which makes it impossible to compare trial results and perform meta-analyses. The absence of a standardized scoring system and paucity of high-quality PWS studies consequently limit (the quality of) the evidence available to clinicians to optimize treatment. As such this problem may have contributed to the lacking improvement in treatment outcomes over the last three decades [10]. Thus, the PWS field would benefit from a single, simple, and easy-to-use clinical assessment protocol, which can preferably be applied both in clinical trials and clinical practice. Inasmuch as it is currently unclear what clinical outcome measure is superior in regard to its measurement properties (i.e., validity, reliability, and responsiveness), we are currently performing a systematic review on the measurement properties of PWS outcome measures. Ideally, a future Delphi study would be organized among a large and relevant international group of stakeholders (including patients and healthcare professionals) to achieve consensus on what outcome constructs should be measured and reported in all PWS treatment research and, subsequently, which instruments should be used to measure these outcomes. If needed, new outcome instruments should be developed, including patient-reported outcomes. Accordingly, another essential part of the process of establishing such a core outcome set is the evaluation of the measurement properties of selected, previously established outcome instruments. Pending these developments, we advise PWS studies to (at least) include a physician-reported score of PWS percentage improvement compatible with quartiles (i.e., the most prevalent scoring system at the top in Fig 4) until consensus on this topic is reached and to report the frequency of each individual outcome category.

The methodological quality of prospective PWS studies could be further improved by consistent implementation of blinded, independent, and experienced evaluators, ensuring sufficient follow-up time, and treatment randomization with inclusion of control groups (e.g., a split-face study design). Moreover, it is imperative that photographs used for clinical assessment are taken under standardized conditions (i.e., using identical camera settings, patient positioning, and lighting). Because of the considerable effects on erythema patients should also be comfortable and stay sufficiently long (e.g., > 30 minutes) in a temperature-controlled room in order to achieve equilibrium conditions before photographs or other measurements are taken. Study quality would also benefit from systematic collection of data on adverse effects and inclusion of (validated) patient-reported outcomes.

## Conclusions

Outcome measures used in prospective PWS studies are highly heterogeneous, making studies incomparable and hampering evidence-based clinical decision making. The results of this systematic analysis underscore the need for reliable, consensus-based, standardized outcome measures.

## Supporting information

S1 Table Searches performed in MEDLINE, Embase, and CENTRAL. (DOCX)

S2 Table Characteristics of the included studies. (DOCX)

S1 Appendix Modified Downs and Black checklist for assessment of methodological quality. (DOCX)

S2 Appendix Modified Downs and Black checklist scores per study. (XLSX)

S3 Appendix Extracted data for all included studies. (XLSX)

S1 Checklist PRISMA checklist. (DOC)
