# Correction: Clinical outcome measures and scoring systems used in prospective studies of port wine stains: A systematic review

**DOI:** 10.1371/journal.pone.0242527

**Published:** 2020-11-12

**Authors:** The PLOS ONE Staff

S1 Table, S2 Table and S1 Appendix are published as incomplete versions. The publisher apologizes for the error. Please view the correct S1 Table, S2 Table and S1 Appendix below.

## Supporting information

S1 Table Searches performed in MEDLINE, Embase, and CENTRAL. (DOCX)

S2 Table Characteristics of the included studies. (DOCX)

S1 Appendix Modified Downs and Black checklist for assessment of methodological quality. (DOCX)
