# Supplementary material for: Clinical outcome measures and scoring systems used in prospective studies of port wine stains: A systematic review
Source: PLoS One. 2020 Jul 2;15(7):e0235657. doi: 10.1371/journal.pone.0235657 (PMC7332045; doi:10.1371/journal.pone.0235657)
Supplement: S2 Table — (DOCX) [file pone.0235657.s002.docx]

| **Table S2. Study characteristics.** | |
| --- | --- |
| **Characteristic** | **N (%)** |
| All studies | 85 |
| Total number of PWS patients | 3,310 |
| Mean age (weighted),^a^ in years | 23.0 |
| Sex^b^ |  |
| Male | 1,173 (40.3) |
| Female | 1,738 (59.7) |
| Country/continent of first author |  |
| China | 32 (37.6) |
| Europe^c^ | 24 (29.4) |
| USA | 12 (14.1) |
| Japan | 4 (4.7) |
| Turkey | 3 (3.5) |
| Egypt | 2 (2.4) |
| Iraq | 2 (2.4) |
| Iran | 1 (1.2) |
| India | 1 (1.2) |
| Pakistan | 1 (1.2) |
| Israel | 1 (1.2) |
| Australia | 1 (1.2) |
| Treatment modality, No. (% of treatment modalities, N = 112^d^) |  |
| 577-nm HOPSL | 1 (0.9) |
| 585-nm PDL | 17 (15.2) |
| 595-nm PDL | 29 (25.9) |
| 585/590/595/600-nm PDL | 3 (2.7) |
| PDL (wavelength NL) | 1 (0.9) |
| IPL | 10 (8.9) |
| PDT | 18 (16.1) |
| 755-nm Alexandrite | 5 (4.5) |
| 1064-nm Nd:YAG | 5 (4.5) |
| 532-nm Nd:YAG | 6 (5.4) |
| DL | 1 (0.9) |
| ICG + DL | 2 (1.8) |
| 585-nm PDL + rapamycin | 1 (0.9) |
| 585-nm PDL + Er:YAG + rapamycin | 1 (0.9) |
| 595-nm PDL + timolol | 1 (0.9) |
| 595-nm PDL + 5-ALA | 2 (1.8) |
| 595-nm PDL + 1064 nm Nd:YAG | 5 (4.5) |
| PDT + PDL | 1 (0.9) |
| PDL + RF | 1 (0.9) |
| 532-nm Nd:YAG + 1064-nm Nd:YAG | 1 (0.9) |
| Electrosclerotherapy with bleomyicin | 1 (0.9) |
| Minimum follow-up time |  |
| < 2 months | 51 (60.5) |
| 2-3 months | 21 (24.4) |
| > 3 months | 5 (5.8) |
| NL | 8 (9.3) |
| ^a^Characteristic reported in N = 56 and ^b^ N = 72 studies. ^c^Studies performed in the United Kingdom (7), Germany (5), The Netherlands (3), Italy (3), Denmark (2), Sweden (1), Poland (1), France (1), Switzerland (1), and Spain (1). ^d^Some studies compared multiple modalities/light applications. Abbreviations: *5-ALA*, 5-aminolevulinic acid; *DL*, diode laser; *Er:YAG*, erbium-doped yttrium aluminum garnet laser; *HOPSL*, high-power optically pumped semiconductor laser; *ICG,* indocyanine green; *IPL,* intense pulsed light; *Nd:YAG,* neodymium-doped yttrium aluminum garnet; *NL*, not listed; *PDT*, photodynamic therapy; *PWS*, port wine stain; *RF*, radiofrequency. | |
