# Supplementary material for: Correction: Clinical outcome measures and scoring systems used in prospective studies of port wine stains: A systematic review
Source: PLoS One. 2020 Nov 12;15(11):e0242527. doi: 10.1371/journal.pone.0242527 (PMC7660535; doi:10.1371/journal.pone.0242527)
Supplement: S1 Table — (DOCX) [file pone.0242527.s001.docx]

**Table S1. Searches performed in MEDLINE, Embase, and CENTRAL.**

| **MEDLINE** | | |
| --- | --- | --- |
| Database(s): **Ovid MEDLINE(R) and Epub Ahead of Print, In-Process & Other Non-Indexed Citations and Daily**1946 to May 01, 2020 Search Strategy: **2020-05-04** | | |
| **#** | **Searches** | **Results** |
| 1 | port-wine stain/ | 989 |
| 2 | ((port win* or portwin*) adj6 l?esion*).tw,ot,kf. | 136 |
| 3 | (port-win* or portwin*).tw,ot,kf. and (exp hemangioma/ or exp angiomatosis/ or vascular malformations/ or capillaries/ or (PWS or stain* or birthmark* or mark or marks or n?evus or n?evi or h?emangiom* or angiom* or malform* or anomal* or SWS or Sturge or Weber or facial or capillar*).tw,ot,kf.) | 1582 |
| 4 | (PWS* adj3 (birthmark* or birth-mark* or n?evus or n?evi or h?emangiom* or angiom* or malformat* or capillar*)).tw,kf. | 72 |
| 5 | ((n?evus or n?evi) and (flamm?eus or vinos*)).tw,ot,kf. | 320 |
| 6 | (vascular adj (ne?vus or n?evi)).tw,ot,kf. | 124 |
| 7 | (birthmarks or vascular birthmark*).ti. | 136 |
| 8 | ((capillar* adj4 malformat*).tw,kf. or (capillaries/ and vascular malformations/)) and (laser* or video* or PDL or photo*).mp. | 162 |
| 9 | ((facial or face or head or neck or lip or lips or trunk or arm or arms or leg or legs or skin or derm* or cutaneous*) adj3 (capillar* adj2 malformat*)).tw,kf. | 129 |
| **10** | **or/1-9 [PWS]** | **2402** |
| 11 | exp animals/ not humans/ | 4695034 |
| **12** | **10 not 11 [ human PWS ]** | **2362** |
| **13** | **limit 12 to yr="2005 -Current" [human PWS -studies >2005]** | **1222** |
| **14** | **remove duplicates from 13 [human PWS -studies >2005 -deduplicated ]** | **1220** |
| 15 | exp guideline/ or exp Evidence-Based Practice/ or exp Databases, Bibliographic/ or meta-analysis/ or (meta analy* or metaanaly* or meta?analy*).tw,kf. or ((systematic* adj3 (review or literature or evidence or search*)) or ((summari* or review) adj3 evidence) or (search* adj12 (literature* or ((electronic or medical or biomedical) adj3 database*) or exhaustive)) or medline or pubmed or embase or (CENTRAL and cochrane) or "Central Register of Controlled Trials").tw. or (cochrane or clinical evidence or EBM).jw. [secondary study filter] | 532357 |
| **16** | **14 and 15 [PWS secondary studies >2005]** | **22** |
| 17 | (exp clinical trial/ or comparative study/ or random allocation/ or cohort studies/ or exp longitudinal studies/ or (groups or subgroup*).ab. or (randomi?ed or placebo* or randomly or (random adj2 allocated) or ((random* or controlled) adj2 study) or trial or ((singl* or doubl* or treb* or tripl*) adj (blind*3 or mask*3)) or cohort* or prospectiv* or longitudinal* or follow-up or enroll* or open label).tw,ot,kf.) not ((review or editorial or letter or comment).pt. not (comparative study.pt. or exp cohort studies/ or exp controlled clinical trial/)) [RCT & prospective study-filter] | 5868319 |
| **18** | **14 and 17 [ human PWS - RCTs & prospective studies >2005 ]** | **286** |

| **Embase** | | |
| --- | --- | --- |
| Database(s): **Embase Classic+Embase**1947 to 2020 May 01 Search Strategy: **2020-05-04** | | |
| **#** | **Searches** | **Results** |
| 1 | nevus flammeus/ | 2716 |
| 2 | ((port win* or portwin*) adj6 l?esion*).tw,ot,kw. | 180 |
| 3 | (port-win* or portwin*).tw,ot,kw. and (angioma/ or exp hemangioma/ or congenital blood vessel malformation/ or capillary/ or (PWS or stain* or birthmark* or mark or marks or n?evus or n?evi or h?emangiom* or angiom* or malform* or anomal* or SWS or Sturge or Weber or facial or capillar*).tw,ot,kw.) | 2247 |
| 4 | (PWS* adj3 (birthmark* or birth-mark* or n?evus or n?evi or h?emangiom* or angiom* or malformat* or capillar*)).tw,ot,kw. | 105 |
| 5 | ((n?evus or n?evi) and (flamm?eus or vinos*)).tw,ot,kw. | 569 |
| 6 | (vascular adj (ne?vus or n?evi)).tw,ot,kw. | 168 |
| 7 | (birthmarks or vascular birthmark*).ti. | 159 |
| 8 | ((capillar* adj4 malformat*).tw,kw. or (capillaries/ and congenital blood vessel malformation/)) and (laser* or video* or PDL or photo*).mp. | 303 |
| 9 | ((facial or face or head or neck or lip or lips or trunk or arm or arms or leg or legs or skin or derm* or cutaneous*) adj3 (capillar* adj2 malformat*)).tw,kw. | 211 |
| **10** | **or/1-9 [PWS]** | **3950** |
| 11 | (animal.hw. or nonhuman/) not human/ | 6473985 |
| **12** | **10 not 11 [ human PWS ]** | **3864** |
| **13** | **limit 12 to yr="2005 -Current" [human PWS -studies >2005]** | **2073** |
| **14** | **remove duplicates from 13 [human PWS -studies >2005 -deduplicated ]** | **2035** |
| **15** | **14 not medline.cr. [ human PWS -deduplicated - EMBASE records only ]** | **1849** |
| 16 | meta analysis/ or "systematic review"/ or (meta analy* or metaanaly* or meta?analy*).tw,kw. or ((systematic* adj3 (review or literature or evidence or search*)) or ((summari* or review) adj3 evidence) or ((search* adj12 (literature* or ((electronic or medical or biomedical) adj3 database*) or exhaustiv*)) or medline or pubmed or embase or psychinfo or (CENTRAL and cochrane) or "Central Register of Controlled Trials")).tw. or (cochrane or clinical evidence or EBM).jw. [secondary study-filter] | 589972 |
| **17** | **15 and 16 [PWS secondary studies >2005]** | **39** |
| 18 | (exp clinical trial/ or controlled study/ or comparative study/ or multicenter study/ or open study/ or cohort analysis/ or longitudinal study/ or prospective study/ or follow-up/ or (groups or subgroup*).ab. or (randomi?ed or placebo* or randomly or (random adj2 allocated) or ((random* or controlled) adj2 study) or trial or ((singl* or doubl* or treb* or tripl*) adj (blind*3 or mask*3)) or cohort* or prospectiv* or longitudinal* or follow-up or enroll* or open label).tw,ot,kw.) not ((review.pt. not (randomized controlled trial/ or controlled clinical trial/ or comparative study/ or cohort analysis/ or longitudinal study/ or prospective study/ or follow-up/)) or (editorial or letter or book or book series or conference review or proceeding or report or trade journal).pt.) [prospective studies-filter] | 12257601 |
| **19** | **15 and 18 [ PWS - RCTs & prospective studies >2005 ]** | **559** |

| **CENTRAL** | | |
| --- | --- | --- |
| Search strategy: **2020-05-04** | | |
| **#** | **Searches** | **Results** |
| #1 | port-wine or portwine | 126 |
| #2 | (PWS near/3 (birthmark* or birth-mark* or naevus or naevi or nevus or nevi or hemangiom* or haemangiom* or angiom* or malformat* or capillar*)) | 5 |
| #3 | (n*vus or n*vi) and (flammeus or vinos*) | 33 |
| #4 | vascular next (nev* or naev*) | 1 |
| #5 | (birthmarks or (vascular NEXT birthmark*)):ti | 7 |
| #6 | (capillar* NEAR/4 malformat*) and (laser* or video* or PDL or photo*) | 15 |
| #7 | ((facial or face or head or neck or lip or lips or trunk or arm or arms or leg or legs or skin or derm* or cutaneous*) NEAR/3 (capillar* NEAR/2 malformat*)) | 2 |
| #8 | #1 or #2 or #3 or #4 or #5 or #6 or #7 | 138 |
| #9 | #8 in Trials | 137 |
| **#10** | **#9 with Publication Year from 2005 to 2020** | **109** |
